# Novel inhibitor candidates of TRPV2 prevent damage of dystrophic myocytes and ameliorate against dilated cardiomyopathy in a hamster model

**DOI:** 10.18632/oncotarget.24449

**Published:** 2018-02-08

**Authors:** Yuko Iwata, Yoshimi Katayama, Yasushi Okuno, Shigeo Wakabayashi

**Affiliations:** ^1^ Departments of Molecular Physiology and Clinical Research, National Cerebral and Cardiovascular Center Research Institute, Suita, Osaka, Japan; ^2^ Pharmacological Research Laboratories, Drug Safety Testing Center Co., Ltd., Higashimatsuyama, Saitama, Japan; ^3^ Department of Clinical System Onco-Informatics, Graduate School of Medicine, Kyoto University, Kyoto, Japan; ^4^ Departments of Molecular Physiology and Cardiac Physiology, National Cerebral and Cardiovascular Center Research Institute, Suita, Osaka, Japan; ^5^ Present affiliation: Biological Research Laboratories, Nissan Chemical Industries, Ltd, Shiraoka, Saitama, Japan; ^6^ Present affiliation: Department of Pharmocology, Osaka Medical Collage, Takatsuki, Osaka, Japan

**Keywords:** cardiomyopathy, muscular dystrophy, Ca2^+^ influx, Ca2^+^-permeable channel, therapy, Gerotarget

## Abstract

Transient receptor potential cation channel, subfamily V, member 2 (TRPV2) is a principal candidate for abnormal Ca^2+^-entry pathways, which is a potential target for therapy of muscular dystrophy and cardiomyopathy. Here, an *in silico* drug screening and the following cell-based screening to measure the TRPV2 activation were carried out in HEK293 cells expressing TRPV2 using lead compounds (tranilast or SKF96365) and off-patent drug stocks. We identified 4 chemical compounds containing amino-benzoyl groups and 1 compound (lumin) containing an ethylquinolinium group as candidate TRPV2 inhibitors. Three of these compounds inhibited Ca^2+^ entry through both mouse and human TRPV2, with IC_50_ of less than 10 μM, but had no apparent effect on other members of TRP family such as TRPV1 and TRPC1. Particularly, lumin inhibited agonist-induced TRPV2 channel activity at a low dose. These compounds inhibited abnormally increased Ca^2+^ influx and prevented stretch-induced skeletal muscle damage in cultured myocytes from dystrophic hamsters (J2N-k). Further, they ameliorated cardiac dysfunction, and prevented disease progression *in vivo* in the same J2N-k hamsters developing dilated cardiomyopathy as well as muscular dystrophy. The identified compounds described here are available as experimental tools and represent potential treatments for patients with cardiomyopathy and muscular dystrophy.

## INTRODUCTION

Dilated cardiomyopathy (DCM) is a severe disorder characterised by ventricular dilation and cardiac dysfunction [1–3]. A subset of familial DCM is caused by mutations in genes encoding components of the dystrophin-glycoprotein complex [4–6], a multi-subunit complex [5, 7, 8] spanning the sarcolemma that links the extracellular matrix to the actin cytoskeleton [9]. Disruption of the dystrophin-glycoprotein complex can significantly compromise membrane integrity and stability during muscle contraction/relaxation and reduce myocyte survival. Enhanced susceptibility to muscle damage is observed in dystrophic animals, such as dystrophin-deficient *mdx* mice and δ-sarcoglycan (SG)-deficient J2N-k hamsters, which exhibit cardiac and skeletal abnormalities similar to those observed in human patients with Duchenne or limb-girdle muscular dystrophy.

Chronic elevation in cytosolic Ca^2+^ concentration ([Ca^2+^]_i_) beneath the sarcolemma and within other cellular compartments has been reported in skeletal muscle fibres and cultured myotubes derived from Duchenne muscular dystrophy patients and *mdx* mice [10–12]. [Ca^2+^]_i_ in muscle fibre cells is regulated by multiple Ca^2+^-permeable channels, Ca^2+^ pumps, and transporters in the plasma membrane and sarcoplasmic reticulum, among which sarcolemmal Ca^2+^-permeable channels (also known as Ca^2+^-specific leak channels) and mechanosensitive, nonselective cation channels contribute to abnormal Ca^2+^ handling in dystrophic myocytes [13, 14]. We previously showed that δ-SG-deficient myocytes are highly susceptible to mechanical stretch and enhanced Ca^2+^ influx via the stretch-activated nonselective Ca^2+^ channel [15] and identified transient receptor potential cation channel, subfamily V (vanilloid), member 2 (TRPV2) as a candidate factor in Ca^2+^ entry pathways whose activation results in perturbation of Ca^2+^ handling and subsequent muscular degeneration [16]. The critical role of TRPV2 in muscular dystrophy was demonstrated using a dominant-negative strategy [17, 18]. Furthermore, sarcolemmal staining of TRPV2 was increased in heart cells from cardiomyopathic J2N-k hamsters. In addition, TRPV2 channel activity was enhanced in J2N-k cardiomyocytes, as evidenced by high [Ca^2+^]_i_ and the observed 2-aminoethoxydiphenylborate (2-APB)-induced increase in [Ca^2+^]_i_ [19]. Similar increased sarcolemmal staining of TRVP2, as well as heart failure, was observed [19] in studies using murine models of DCM (doxorubicin-induced DCM mice and sugar chain abnormal 4C30 DCM mice [20]) and in human patients with idiopathic DCM [19]. We observed that reducing TRPV2 activity was an effective therapeutic strategy for muscular dystrophy and cardiomyopathy [17, 19]. Moreover, we found that anti-allergy agent N-[3,4-dimethoxycinnamonyl]-anthranilic acid (tranilast) inhibited Ca^2+^ entry through TRPV2 and ameliorated muscle degeneration [19]. 1-[β-[3-(4-methoxyphenyl)propoxy]-4-methoxyphenethyl]-1H-imidazole (SKF96365) has also been reported to inhibit TRPV family channels [21]. However, because the effective doses of known TRPV2 inhibitors are high (>10–100 μM), while the drugs are also relatively unselective, more potent and specific TRPV2 inhibitors are needed to confirm whether TRPV2 is an effective drug target for the treatment of patients with DCM and related disorders.

**Figure 1:**
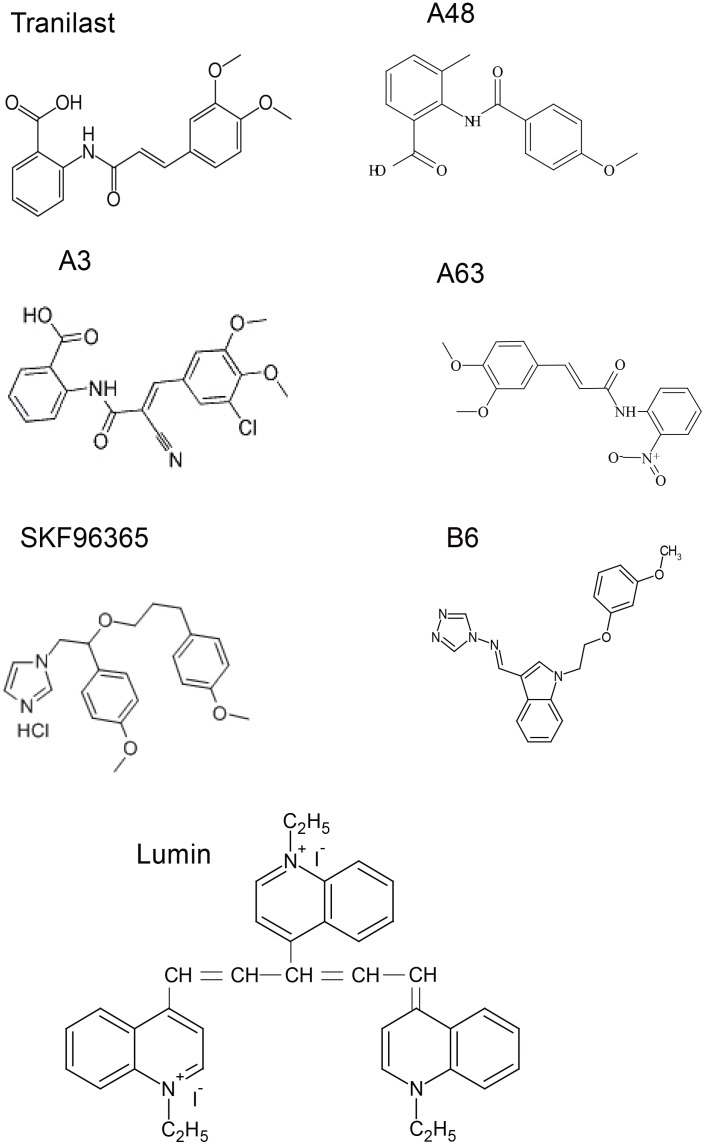
Structures of chemical compounds

Here, a high throughput assay was performed to screen a chemical library for potential TRPV2 inhibitors. The TRPV2 agonist 2-APB, which induced a large increase in TRPV2 activity and [Ca^2+^]_i_ under weak acidic conditions, was used to screen candidate compounds. Several TRPV2 inhibitors were identified using tranilast and SKF96365 (Figure 1) as lead compounds. The beneficial effects of the newly identified TRPV2 inhibitors were assessed in dystrophic/cardiomyopathic hamsters.

## RESULTS

### High throughput Ca^2+^ measurements for TRPV2 inhibitor screening

For TRPV2 inhibitor screening, we used a cell-based assay system to monitor TRPV2-dependent increases in [Ca^2+^]_i_. We initially assessed several reported TRPV2 agonists, including 2-APB, probenecid, and cannabidiol; however, the Ca^2+^ responses elicited by these agonists were too small for the purpose of drug screening. Therefore, we aimed to identify conditions suitable for detecting the Ca^2+^-response elicited by 2-APB, a widely used TRPV agonist.

**Figure 2:**
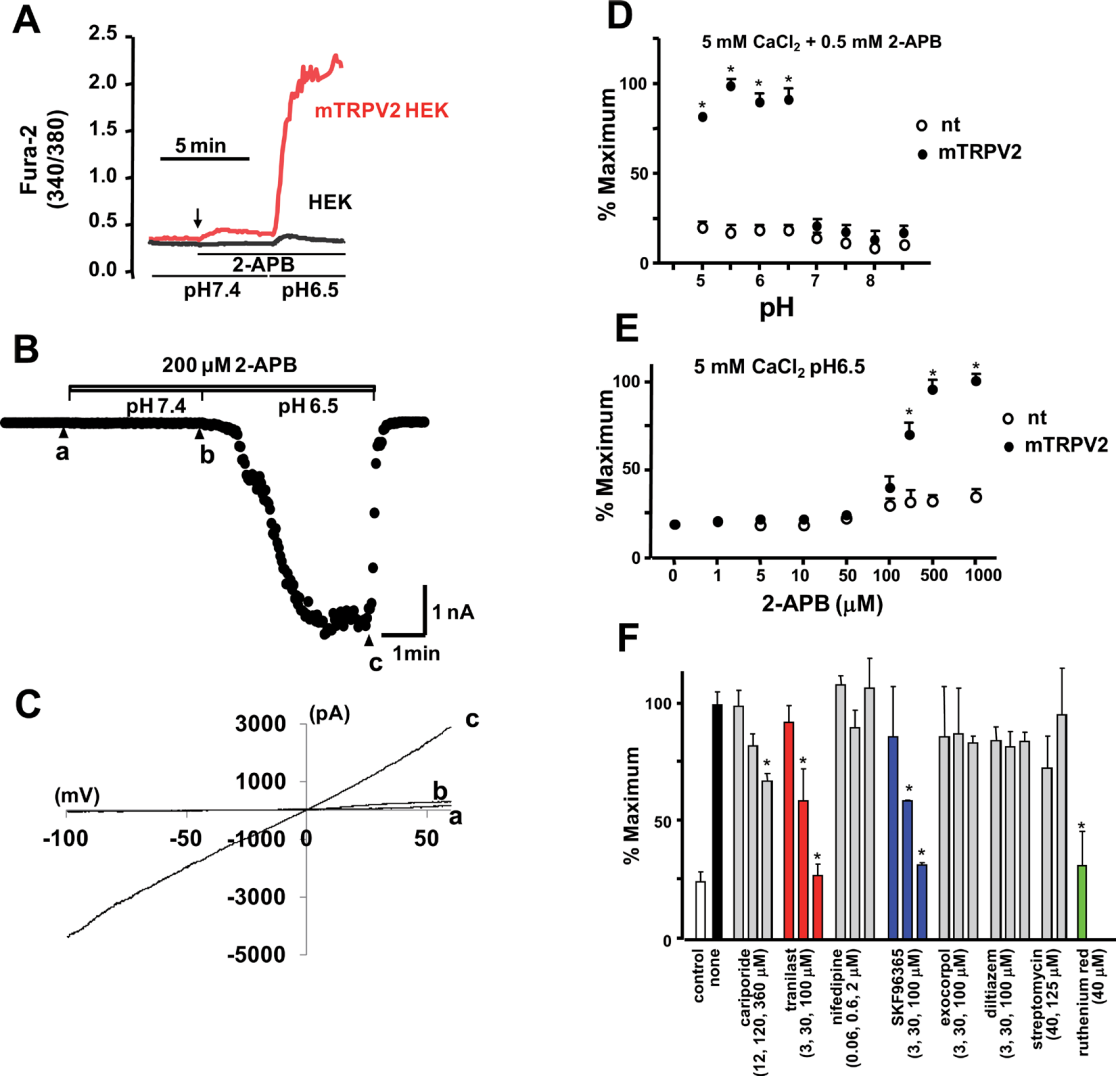
Effects of extracellular pH and 2-APB on Ca^2+^ response and TRPV2 channel activity in HEK293 cells expressing TRPV2 (**A**) Non-transfected (HEK) or mTRPV2-transfected HEK293 cells were loaded with 4 μM fura2-AM and maintained in BSS containing low CaCl_2_ (0.5 mM) at pH 7.4. Cells were stimulated with medium (pH 7.4) containing CaCl_2_ (5 mM) and 2-APB (500 μM) (arrow), followed by exposure to low pH (6.5) medium containing CaCl_2_ and 2-APB. (**B**) Traces of currents elicited by the ramp protocol. The mTRPV2 currents were recorded with the holding potential at −60 mV. The patch clamped cells were perfused with medium (pH 7.4) containing 200 μM 2-APB and then switched to the low pH (6.5) medium. The current values at −100 mV with an every-2-second ramp protocol were plotted. (**C**) Current-voltage relationship. The time points for the traces shown as a, b, and c are shown in (B). (**D**) Extracellular pH-dependence of the Ca^2+^ response measured by the fluorescence microplate reader. Cells were initially incubated in BSS containing low CaCl_2_ (0.5 mM) at pH 7.4 and then switched to BSS containing high CaCl_2_ (5 mM) and 2-APB (0.5 mM) at the indicated pH values. Fura-2-AM fluorescence was measured at excitation wavelengths of 340 and 380 nm. The fluorescence ratio measured at 340/380 nm was normalized by the maximal value and plotted. The data represent mean ± SD values (*n* = 5/group); ^*^P < 0.05. (**E**) Dependence of the Ca^2+^ response on 2-APB concentration. The medium was switched to BSS at an acidic pH (6.5) containing high CaCl_2_ and various concentrations of 2-APB. The data represent mean ± SD values (*n* = 5/group); ^*^P < 0.05. (**F**) Cells were stimulated with 2-APB (0.3 mM) and CaCl_2_ (5 mM) at pH 6.5 in the absence or presence of each compound at the indicated concentrations. The fluorescence ratio at 340/380 nm was normalized by the value measured with 2-APB alone. Open bar, no 2-APB (control). The data represent mean ± SD values (*n* = 5/group); ^*^P < 0.05.

HEK293 cells expressing mouse TRPV2 (mTRPV2) and non-transfected HEK293 cells were loaded with fura-2-AM and placed in low CaCl_2_ medium (0.5 mM). When the medium was replaced with high Ca^2+^ medium (5 mM) at neutral pH (7.4) containing TRPV agonist 2-APB, the increase in [Ca^2+^]_i_ was very small (Figure 2A). However, when cells were perfused with weak acidic medium (pH 6.5), a large increase in [Ca^2+^]_i_ was detected in TRPV2-expressing HEK293 cells. We tested whether low pH-dependent 2-APB-induced activation of TRPV2 was also detected in the TRPV2 current measured by the whole cell patch clamp technique. Consistent with the response to Ca^2+^, low pH dramatically activated TRPV2 current in the presence of 2-APB (Figure 2B). The observed pH-dependent TRPV2 activation occurred at all tested voltages (Figure 2C), indicating a voltage-independent effect. These results indicate that TRPV2 activation by 2-APB is enhanced at low pH.

For the drug screening, we designed a method involving measurement of the Ca^2+^ response using a 96-well high-throughput plate-reader. Cells were placed in low CaCl_2_ (0.5 mM) medium, which was replaced with high CaCl_2_ medium (5 mM) with or without the TRPV agonist 2-APB and adjusted to various pH values. While no effects were observed at neutral extracellular pH, a marked increase in [Ca^2+^]_i_ was detected under acidic conditions (pH < 7.0), concomitant with a pH drop from 7.0 to 6.5 (Figure 2D). The dependence of the Ca^2+^ response on the concentration of 2-APB was evaluated at 5 mM CaCl_2_/pH 6.5. [Ca^2+^]_i_ increased upon addition of 2-APB in the range of 0.3–1.0 mM (Figure 2E). Thus, a screening for drugs effective in acidic medium containing 2-APB and a high (extracellular) CaCl_2_ was carried out.

### TRPV2 inhibitor screening

**Figure 3:**
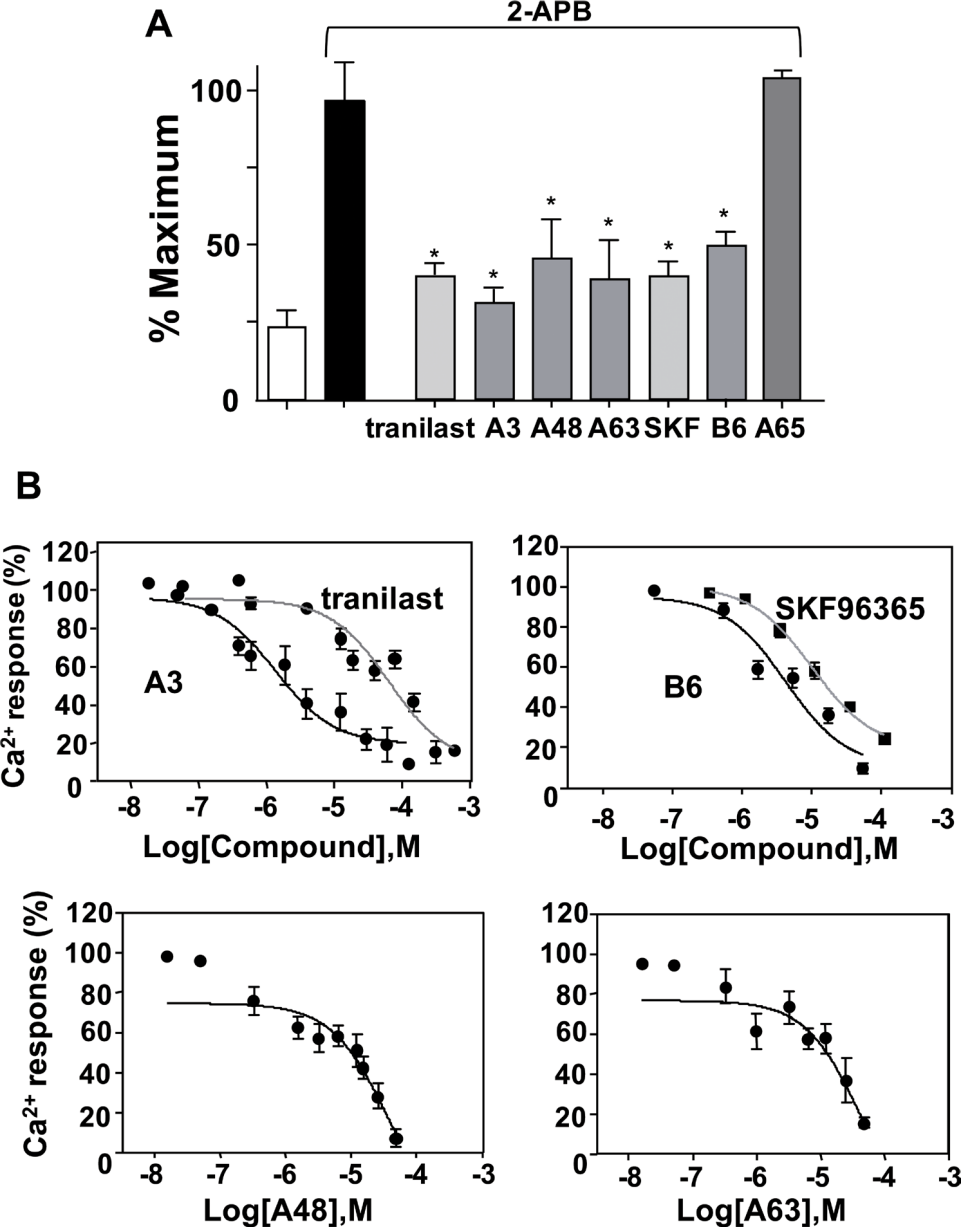
Effects of inhibitor compounds on 2-APB-induced Ca^2+^ increase in HEK293 cells expressing mTRPV2 (**A**) Cells cultured in 96 well plates were stimulated with 2-APB (0.3 mM) and CaCl_2_ (5 mM) at pH 6.5 in the absence (solid bar) or presence of each candidate inhibitor (15 μM), tranilast (100 μM), or SKF96365 (100 μM). Open bar, no 2-APB or inhibitor. Data are shown as mean ± SD (*n* = 5). ^*^P < 0.05. (**B**) Concentration-dependence of inhibition of the 2-APB-induced Ca^2+^ response. The 2-APB-induced increase in fluorescence at 340/380 nm was normalised to that measured in the absence of the inhibitors. Data are shown as mean ± SD (*n* = 5).

Drugs known to inhibit ion transport proteins were tested using the high throughput screening system. Exposure to inhibitors of the Na^+^/H^+^ exchanger (cariporide), stretch-activated channels (streptomycin), and voltage-gated Ca^2+^ channels (diltiazem) at concentrations up to 100 μM did not inhibit the 2-APB-induced increase in [Ca^2+^]_i_ in HEK293 cells expressing mTRPV2 (Figure 2F). The nonselective cation channel blockers tranilast and SKF96365 [22, 23] had an inhibitory effect on the 2-APB-induced increase in [Ca^2+^]_i_ in HEK293 cells expressing mTRPV2 at high doses (IC_50_ ≥ 10 μM). Using tranilast and SKF96365 as lead compounds, 350 drug candidates were selected from the chemical library and classified as series A or B, respectively. The 350 drug candidates were screened for the ability to inhibit Ca^2+^ entry through TRPV2 at a low concentration. A3, A48, A63, and B6 (See Figure 1 and materials and methods for chemical structure) inhibited Ca^2+^ entry in mTRPV2-expressing HEK293 cells (Figure 3A) and were more potent than tranilast and SKF96365 (IC_50_ < 2 μM for A3; IC_50_ < 10 μM for A48 and A63; IC_50_ approximately 4 μM for B6) (Figure 3B). Other tested chemical compounds, including A65 and B33 (data not shown), had negligible effects on the 2-APB-induced increase in [Ca^2+^]_i_ (Figure 3A).

### Specificity of TRPV2 inhibitors

**Figure 4:**
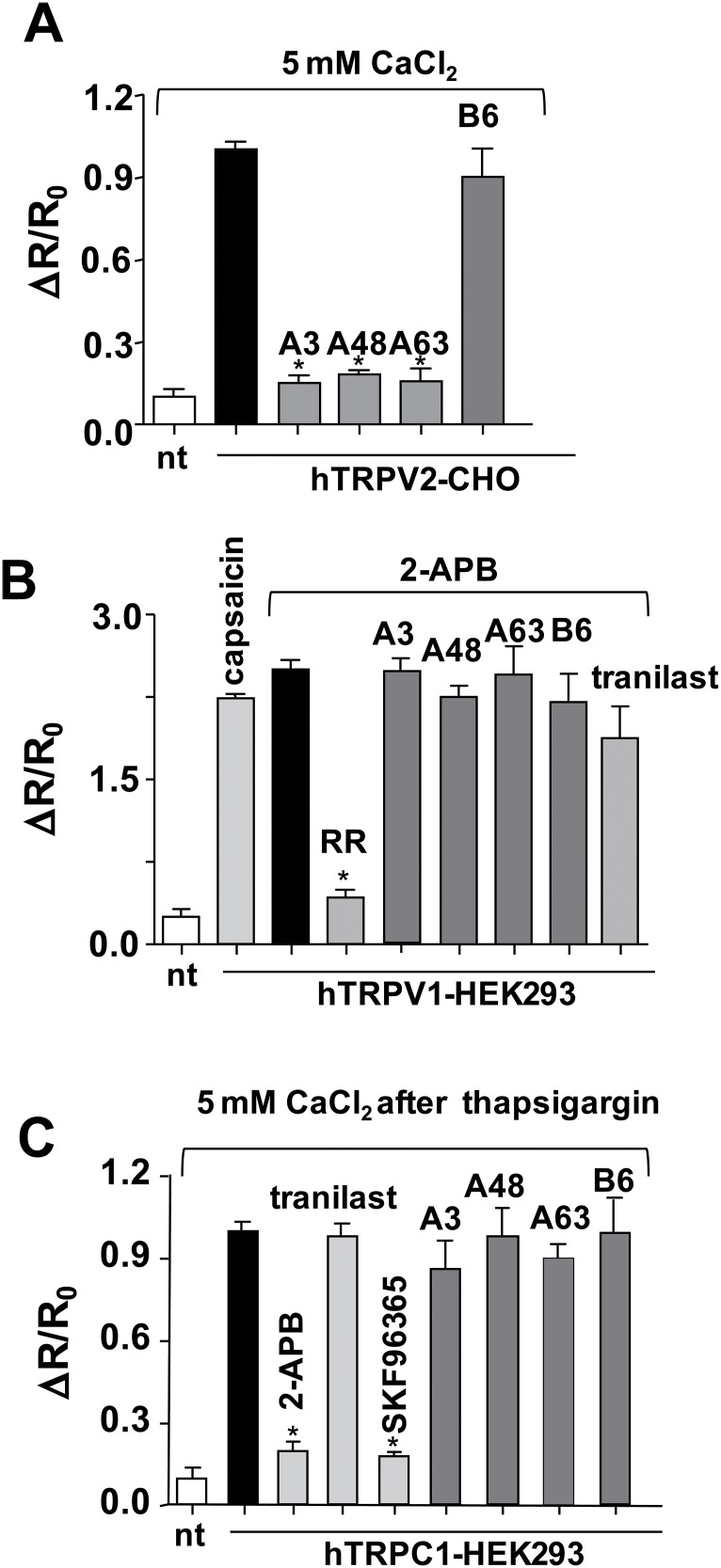
Effects of inhibitor compounds on the Ca^2+^ response of cells expressing hTRPV1, hTRPV2, or hTRPC1 (**A**) Effect of compounds on CaCl_2_ (5 mM)-induced peak fluorescence ratio in non-transfected (nt) or hTRPV2-transfected CHO cells. The extracellular Ca^2+^-dependent increase in the ratio of fura-2 fluorescence intensity at 340/380 nm (ΔR, 2 min after addition of chemicals) was plotted as ΔR/R_0_ after normalising to the initial fluorescence ratio (R_0_). Concentration of A3, A48, A63, and B6: 15 μM. Solid bar, no inhibitor. (**B**) Effect of compounds on 2-APB (1 mM)/high extracellular CaCl_2_-induced peak fluorescence ratio in nt or hTRPV1-transfected HEK293 cells. The 2-APB/high extracellular Ca^2+^-dependent increase (ΔR) in the fluorescence intensity ratio was measured 2 min after addition of 2-APB. Concentrations: capsaicin and tranilast, 100 μM; ruthenium red (RR), 40 μM; A3, A48, A63, and B6, 15 μM. (**C**) Effect of compounds on store-operated Ca^2+^ entry in nt or hTRPC1-transfected HEK293 cells. Cells were treated with the endoplasmic reticulum Ca^2+^ pump inhibitor thapsigargin and exposed to CaCl_2_ and the indicated compounds. ΔR was measured 2 min after the addition of CaCl_2_. Concentrations: 2-APB, 10 μM; tranilast and SKF96365, 100 μM; A3, A48, A63, and B6, 15 μM. Data are shown as mean ± SD (*n* = 5).

Unlike mTRPV2, human TRPV2 (hTRPV2) is unresponsive to 2-APB [21]. Therefore, to determine whether the identified chemical compounds inhibit other human TRP family members, the increase in [Ca^2+^]_i_ induced by a high concentration (5 mM) of extracellular CaCl_2_ was measured in CHO cells expressing hTRPV2 using a fluorescence ratio monitoring system. In this experiment, we used CHO cells instead of HEK293 cells, because the Ca^2+^ response induced by high (extracellular) Ca^2+^ is detected relatively easily in CHO cells. Ca^2+^ influx in response to CaCl_2_ exposure was observed in CHO cells expressing either mTRPV2 or hTRPV2 [17]. The observed Ca^2+^ influx in response to CaCl_2_ exposure was markedly inhibited by A3, A48, and A63, but not by B6 (Figure 4A) or B33 (data not shown). The differential effects of B6 on mTRPV2 and hTRPV2 may be explained by subtle, species-specific variations in the primary structure of the channel. The increase in [Ca^2+^]_i_ induced by 2-APB was also observed in cells expressing hTRPV1 and was comparable to that induced by capsaicin, a specific TRPV1 agonist. However, in contrast to TRPV2, the increase in [Ca^2+^]_i_ via TRPV1 was unaffected by exposure to A3, A48, A63, or B6 (Figure 4B). The effects of A3, A48, A63, and B6 on store-operated increases in Ca^2+^ in cells expressing hTRPC1 were examined after the Ca^2+^ store was depleted with thapsigargin in Ca^2+^-free medium and the cells were exposed to high extracellular Ca^2+^ to induce an increase in [Ca^2+^]_i_. 2-APB inhibited the store-operated Ca^2+^ increase in [Ca^2+^]_i_ in these cells, consistent with previous reports that 2-APB is a potent non-specific inhibitor of TRPC family members [24]. While SKF96365 strongly inhibited this response, neither A3, A48, A63, nor B6 had any effect (Figure 4C). These results suggest that A3, A48, and A63 inhibit TRPV2, but not TRPV1 or TRPC1.

### Protective effects of TRPV2 candidate inhibitors against abnormal Ca^2+^ handling and CK release in dystrophic myocytes from dystrophic hamsters (J2N-k)

**Figure 5:**
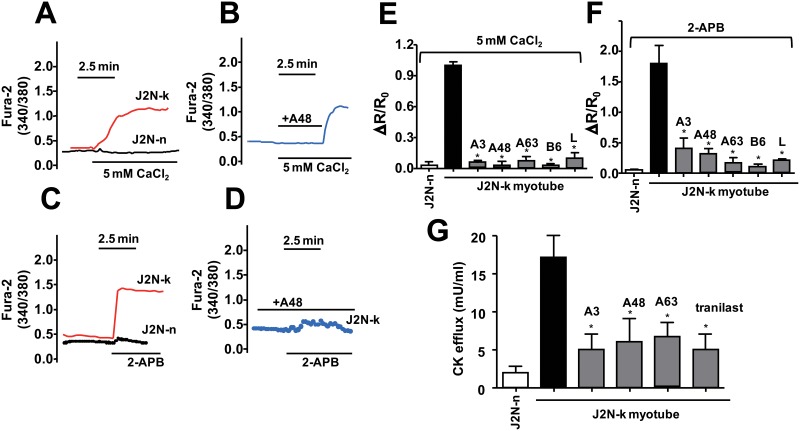
Effect of inhibitor compounds on the Ca^2+^ response in myocytes from hamster skeletal muscles (**A**) Typical traces from ratiometric scanning of fura-2-AM fluorescence in myocytes prepared from wild-type (J2N-n) or dystrophic (J2N-k) hamsters. Myocytes loaded with fura-2-AM were incubated in BSS containing a low concentration of CaCl_2_ (0.5 mM), then exposed to a high concentration of CaCl_2_ (5 mM). The data represent mean values (*n* = 5–10 /group). (**B**) Effect of A48 on the Ca^2+^ response. Myocytes from J2N-k hamsters were exposed to high Ca^2+^ with A48 (15 μM) for 3 min, after which A48 was removed from the medium. (**C**) Myocytes loaded with fura-2-AM were incubated in BSS with a low concentration of CaCl_2_ (0.5 mM), after which the medium was replaced with BSS containing a high concentration of CaCl_2_ (5 mM) and 2-APB (0.5 mM) at pH 6.5. The data represent mean values (*n* = 5–10 /group). (**D**) An experiment similar to that shown in (C) was performed in the presence of A48. (**E**, **F**) Effects of compounds on the CaCl_2_- and 2-APB-induced increases in the relative fura-2 fluorescence ratio (ΔR/R_0_), which was calculated from the fluorescence intensity at 340/380 nm before and after exposure to CaCl_2_ or 2-APB. The concentration of each compound was 15 μM (L, lumin). Data are shown as mean ± SD (*n* = 5). ^*^P < 0.05. (**G**) Stretch-induced CK release from myocytes into the medium in the absence or presence of each drug (15 μM). Data are shown as mean ± SD (*n* = 5). ^*^P < 0.05.

Perfusion with high extracellular Ca^2+^ (5 mM) raised [Ca^2+^]_i_ in dystrophic myocytes from J2N-k, but not in normal myocytes from wild-type J2N-n hamsters (Figure 5A), consistent with previous observations [17]. The extracellular Ca^2+^-induced increase in [Ca^2+^]_i_ in J2N-k myocytes was almost completely abolished by A48 (Figure 5B). A 2-APB-induced increase in [Ca^2+^]_i_ was detected only in J2N-k myocytes (Figure 5C) and was abolished by A48 (Figure 5D). All TRPV2 inhibitors effectively inhibited high (extracellular) Ca^2+^- and 2-APB-induced increases in [Ca^2+^]_i_ in J2N-k myocytes (Figure 5E, 5F). These results suggest that Ca^2+^ influx through TRPV2 is a major contributor to abnormal Ca^2+^ handling observed in J2N-k myocytes. In addition, these results demonstrate that at least 3 (A3, A48, and A63) of the newly identified TRPV2 inhibitors effectively target hamster, mouse, and human TRPV2 isoforms.

To assess the effects of the newly identified TRPV2 inhibitors on mechanical stretch-induced muscle degeneration, J2N-k myocytes were treated with a test agent for 30 min, after which cyclic stretch (20% elongation) was applied for 1 h. CK release was measured as an indicator of muscle damage. Treatment of myocytes with A3, A48, or A63 (30 μM), as well as tranilast (500 μM), reduced stretch-induced CK release from J2N-k myocytes by up to 70% (Figure 5G).

### Protective effects of TRPV2 inhibitor candidates on cardiomyopathy *in vivo*

**Figure 6:**
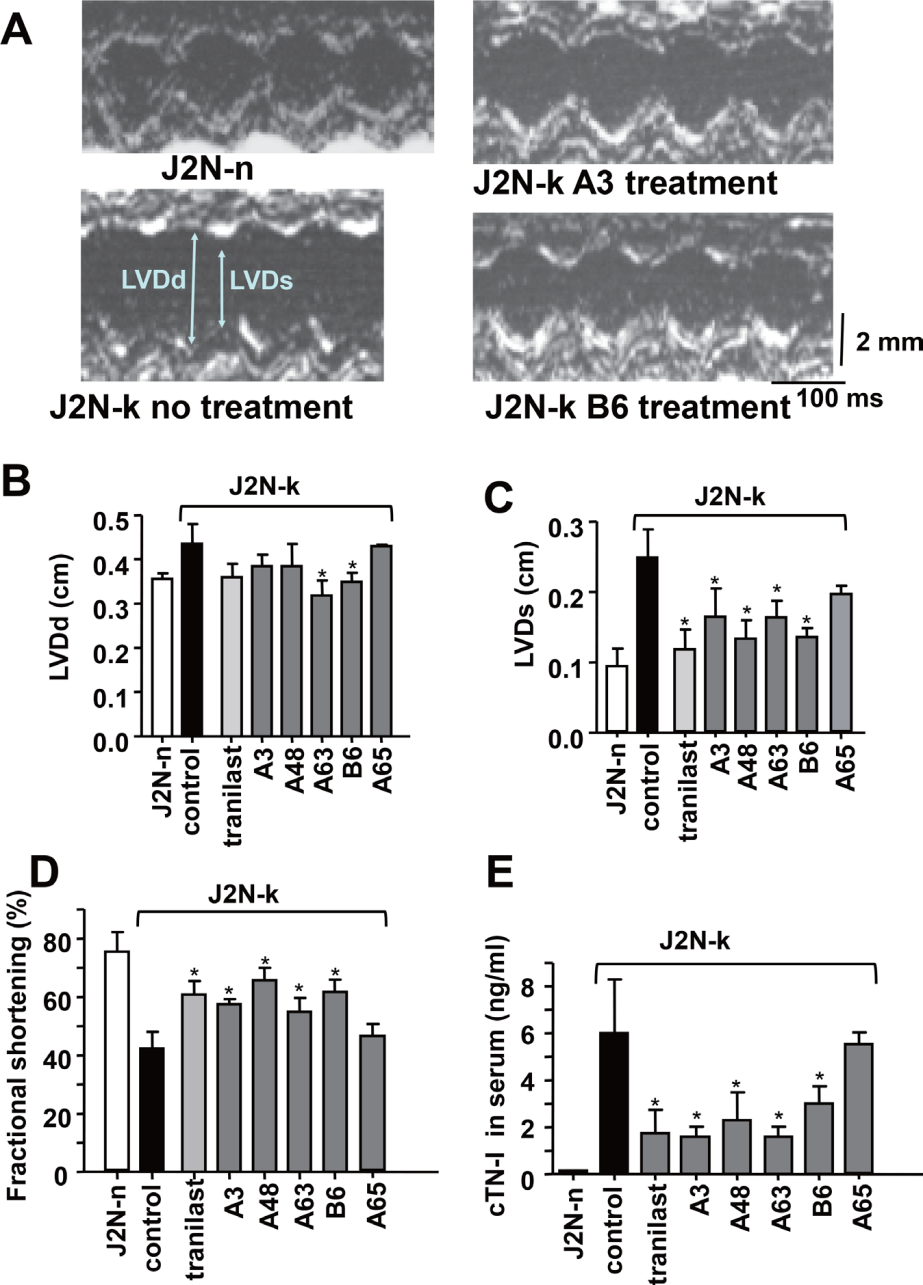
Protective effects of TRPV2 inhibitors against cardiac dysfunction in J2N-k hamsters (**A**) Representative echocardiogram from each group showing left ventricular end diastolic and systolic dimensions (LVDd and LVDs, respectively) in wild-type (J2N-n) and cardiomyopathic (J2N-k) hamsters with or without inhibitor treatment. (**B**–**D**) Effect of inhibitors on LVDd, LVDs, fractional shortening (FS), and (**E**) cTN-I level in the serum. Solid black bar, no inhibitor. Data are shown as mean ± SD (*n* = 4). ^*^P < 0.05 vs. water in J2N-k hamsters.

**Figure 7:**
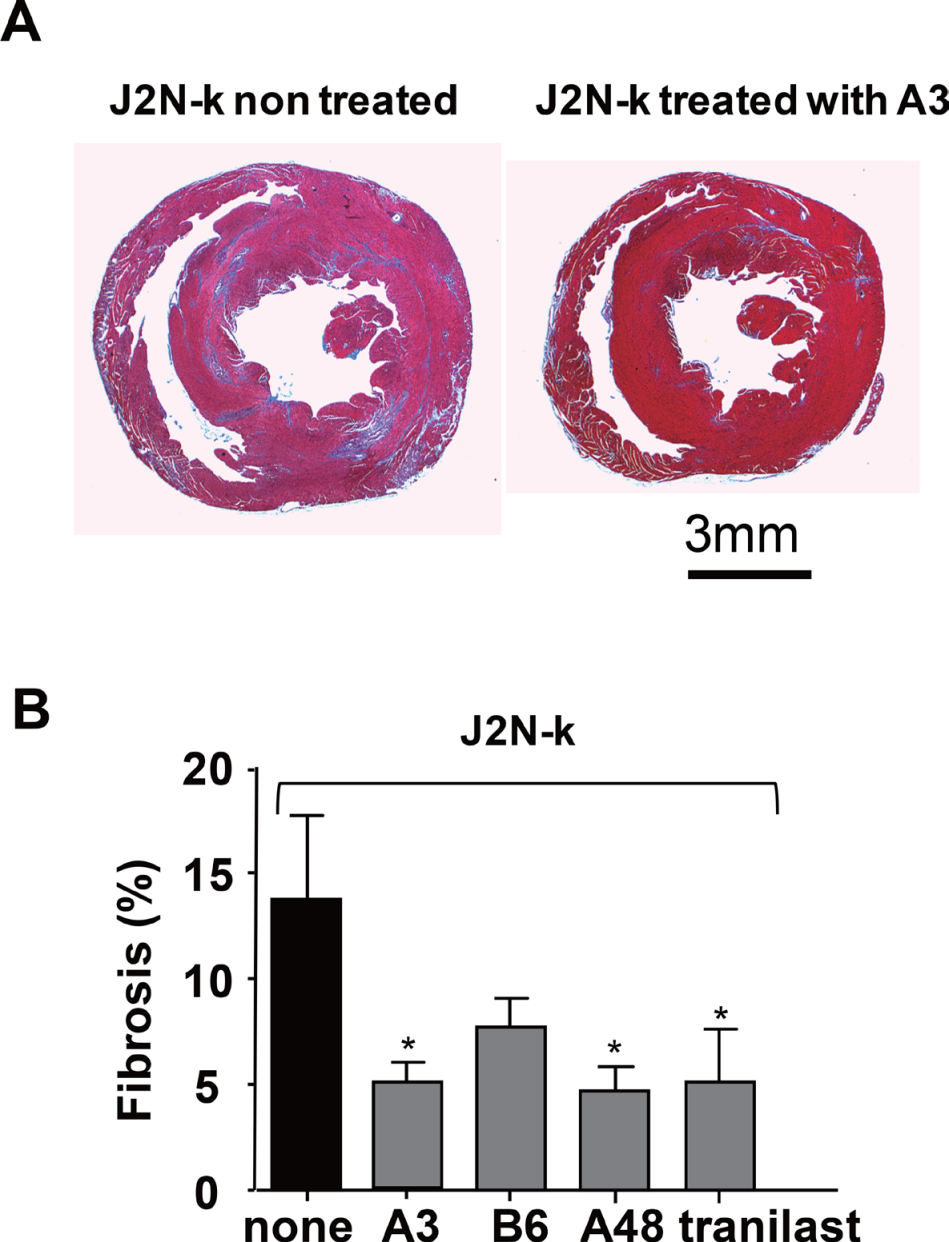
Suppression of fibrosis in the hearts of cardiomyopathic hamsters by TRPV2 inhibitors (**A**) Representative Masson's trichrome staining of transverse heart sections from J2N-k hamsters with or without A3 treatment. (**B**) Quantitative analysis of fibrotic area with or without drug treatment. Data are shown as mean ± SD (*n* = 3–5/group ). ^*^P < 0.05.

To test the efficacy of the newly identified TRPV2 inhibitors *in vivo*, they were administered for 3 weeks to 9-week-old hamsters showing decreased cardiac function, after which echocardiography was performed (Figure 6A). J2N-k hamsters showed left ventricular cavity dilation (Figure 6A). In contrast to small effect on left ventricular end-diastolic dimension (LVDd) (Figure 6B), tranilast, A3, A48, A63, and B6 prevented the increase in left ventricular end-systolic dimension (LVDs) (Figure 6C) and decrease in FS (Figure 6D), while A65 (a negative compound identified by screening) was ineffective. Tranilast, A3, A48, A63, and B6, but not A65, also reduced the serum level of cTN-I, a marker for heart cell damage (Figure 6E). Consistent with the echocardiography results, treatment with A3 prevented fibrosis, as revealed by Masson's trichrome staining (Figure 7A). As summarized in Figure 7B, A3, A48, and tranilast prevented fibrosis.

### Identification of lumin as a TRPV2 inhibitor and its protective effect on cardiomyopathy

**Figure 8:**
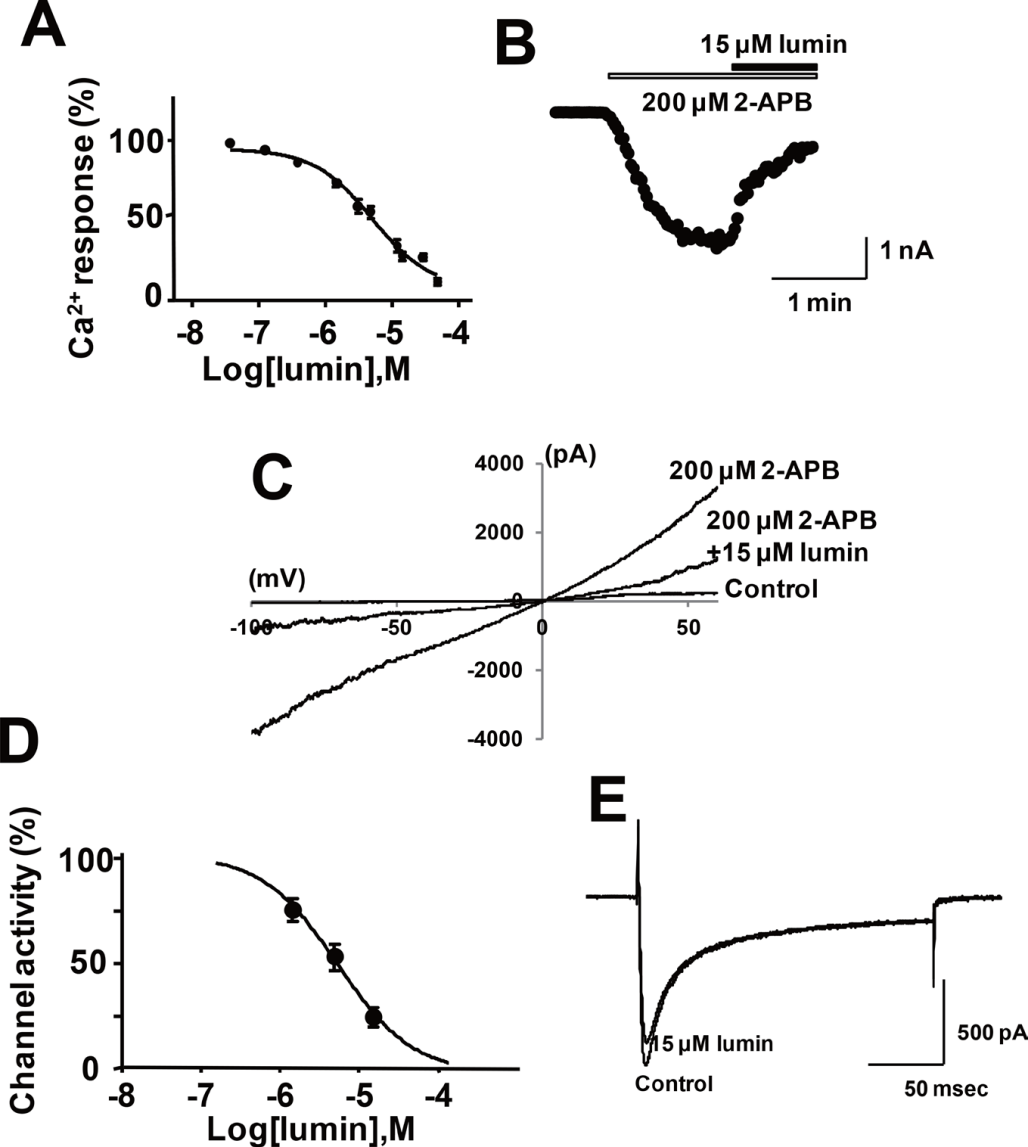
Effect of lumin on the Ca^2+^ response and channel activity in HEK293 cells expressing mTRPV2 (**A**) Inhibition of the 2-APB-induced increase in [Ca^2+^]_i_ in HEK293 cells expressing mTRPV2 by lumin. Cells were loaded with fura-2-AM and stimulated with 2-APB (0.3 mM) in the absence or presence of the indicated concentrations of lumin. The fluorescence ratio was measured by a microplate reader and normalized by the maximal value. Data are shown as mean ± SD (*n* = 5). (**B**) Lumin blocked the mTRPV2 channel. The current values at −100 mV measured by an every-2-second ramp protocol were plotted. After the current activated by 200 μM 2-APB at pH 6.5 became stable, a mixture of 15 μM lumin and 2-APB was applied to the cells. (**C**) Effect of lumin on the current-voltage relationship of 2-APB-evoked current. (**D**) Concentration-dependence of lumin for the inhibition of mTRPV2 current. Data are shown as mean ± SD (*n* = 3–5) (**E**) Effect of lumin (15 μM) on L-type Ca^2+^ channel activity. The current waveforms of the control cells and the cells 11 minutes after application of 15 μM lumin were superimposed. The L-type Ca^2+^ channel currents were elicited by depolarizing pulses of 0 mV for 200 milliseconds from −80 mV.

**Figure 9:**
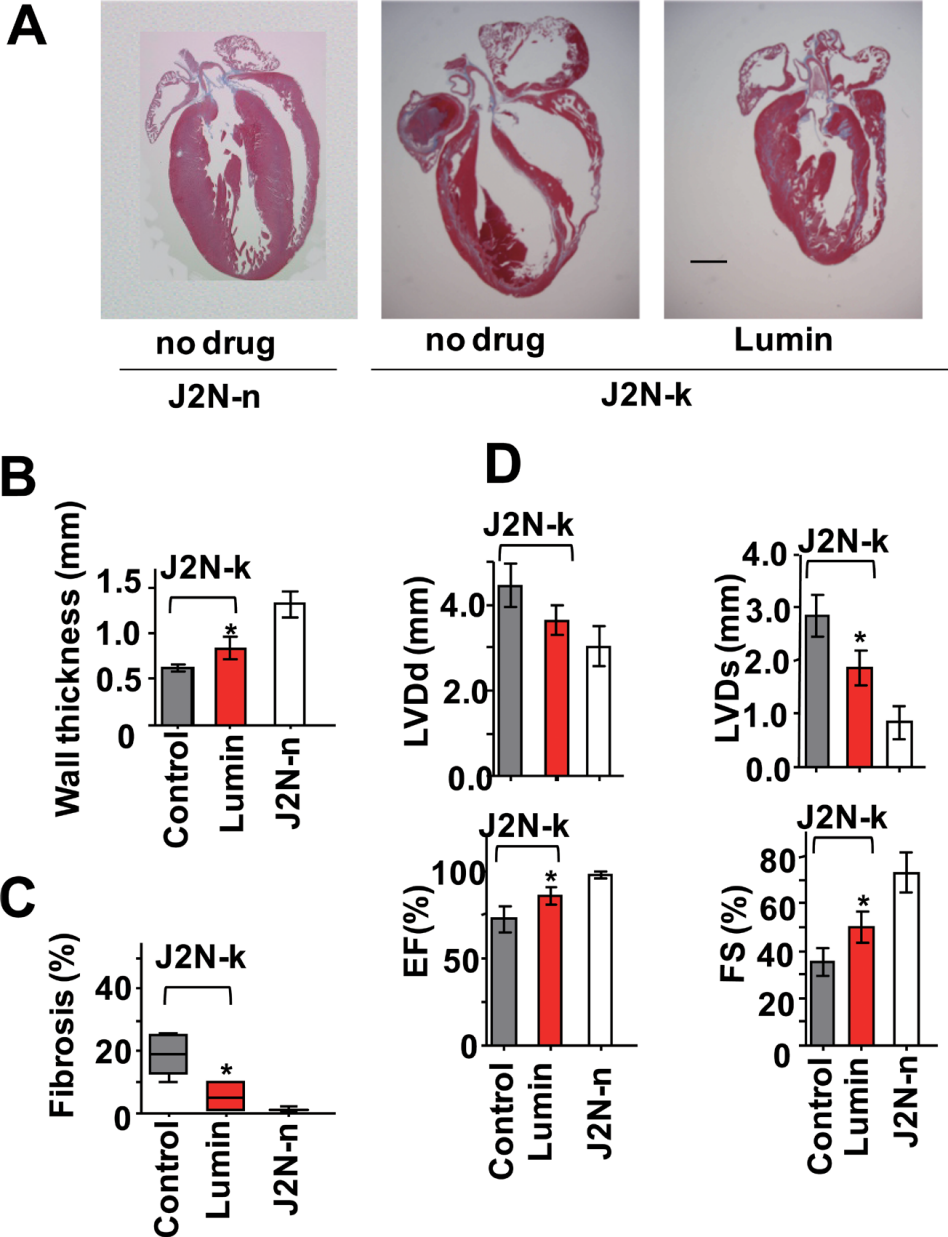
Protective effect of lumin against cardiomyopathy (**A**) Representable Masson's trichrome staining of cardiac sections from wild-type J2N-n and cardiomyopathic J2N-k hamsters administered no compound (control) or lumin (10 mg/kg). Scale bar: 1 mm. (**B** and **C**) Quantitative analysis of septal wall thickness (B) and fibrotic area (C). (**D**) Effect of lumin (10 mg/kg) administration on echocardiographic parameters. Grey bar, no compound (control); EF, ejection fraction; FS, fractional shortening. Data are shown as mean ± SD (*n* = 4–5). ^*^P < 0.05 vs. no compound in J2N-k hamsters.

In parallel with drug screening based on structural similarity with tranilast or SKF96365, we attempted to identify TRPV2 inhibitors by screening various off-patent drugs. A screening of photosensitive dyes yielded lumin [25] as a candidate TRPV2 inhibitor. Lumin inhibited 2-APB-induced Ca^2+^ influx through mTRPV2 in HEK293 cells at a concentration 10-fold lower than the concentration of tranilast required to produce a similar effect (compare Figures 8A and 3B). In addition, lumin inhibited high (extracellular) Ca^2+^ -induced or 2-APB-induced increase in [Ca^2+^]_i_ in dystrophic myotubes from J2N-k hamsters (Figure 5E and 5F). Lumin (15 μM) also reduced the 2-APB-induced current (Figure 8B) at all tested voltages (Figure 8C), the IC_50_ (5 μM) of lumin for the ion current was very similar to the IC_50_ for the Ca^2+^ response (<5 μM) under our experimental conditions. Lumin (15 μM) did not inhibit the L-type current (Figure 8E): 96.6 **±** 8.7 % (*n* = 3) of control at 15 μM lumin. Lumin did not affect the increase in [Ca^2+^]_i_ via TRPV1 or TRPC1 (unpublished observation). When lumin was administered to 9-week-old J2N-k hamsters for 3 weeks, the decrease in wall thickness was partially prevented, fibrotic area was reduced (10 mg/kg dose, Figure 9A–9C), and cardiac dysfunction (increased LVDs, reduced ejection fraction, and decreased fractional shortening) was prevented (Figure 9D).

## DISCUSSION

An assay system developed to measure TRPV2 activation by high throughput screening was used to identify several TRPV2 inhibitors that could be used to treat patients with muscle disorders caused by abnormal Ca^2+^ handling. Besides 2-APB, Lysophospholipids [26], probenecid [27], and cannabidiol [28] have been identified as agents that induce increased [Ca^2+^]_i_ in HEK293 cells expressing TRPV2 [26–28]. Koch [29] and Rubinstein [30] identified probenecid as a potent TRPV2 agonist in cardiomyocytes. We observed a small [Ca^2+^]_i_ increase induced by these agonists in TRPV2-expressing HEK293 cells. However, the Ca^2+^ response was too small for use as a high-throughput screening procedure for the inhibitors. We found that treating cells with 2-APB in a weak acidic medium (pH 6.5) was a reasonably effective way of measuring increases in [Ca^2+^]_i_ mediated by TRPV2. Exposure to weak acidic medium in the presence of 2-APB also dramatically activated the TRPV2 current measured under voltage-clamped conditions using the whole cell patch clamp technique. As previously reported by Chung [31], 2-APB had only a small effect on mTRPV2 under neutral conditions (Figure 2). TRPV1, a TRPV family member sharing 46% identity in the primary amino acid sequence with TRPV2 [32], is directly activated by low external pH. The evidence presented here shows that TRPV2 can be activated by H^+^, although direct activation of TRPV2 by low pH has not yet been reported. The pK_a_ of 2-APB is approximately 10, which means that its amine group is protonated at physiological or lower pH, giving the molecule a net positive charge. Thus, the present results suggest that the H^+^-sensitivity of TRPV2 is due to the existence of a proton-responsive region within the TRPV2 molecule and not to the effect of protonation of 2-APB.

The screening identified several candidate TRPV2 blockers. A3, A48, and A63 were derived from tranilast and had inhibitory potency 2–10-fold higher than that of tranilast (A3 > A48 >A63). A3, A48, and A63 had similar effects on mTRPV2 and hTRPV2. In contrast, B6 was the only compound identified by the screening that was based on SKF96365. Although the inhibitory effect of B6 against mouse TRPV2 was slightly greater than that of SKF96365 (Figure 3B), it had no effect on cells expressing hTRPV2 (Figure 4A). Thus, like 2-APB, the effectiveness of B6 on TRPV2 was species-specific, suggesting that the mechanism of inhibition by B6 may be different from that of tranilast derivatives. Ruthenium red is known as a broad inhibitor of many Ca^2+^-permeable ion channels. In addition, the potassium channel blockers tetraethylammonium, 4-amino-pyridine, and 1-(2-(trifluoromethyl)phenyl)imidazole inhibit TRPV2 activation [21]. However, effective doses of these agents are much higher (100–1000 μM) than those of the candidate inhibitors reported here.

In addition to Ca^2+^ influx via TRPV2, we examined the effects of chemical compounds on TRPV2 channel activity under voltage clamp conditions. We found that lumin effectively blocked TRPV2 channel activity (Figure 8B–8D). However, other inhibitors (A3, A48, A63, and B6) only slightly inhibited TRPV2 channel activity (up to 25% inhibition at 15~30 μM) (data not shown). Thus, the weak effects of these drugs on channel activity were different from their relatively strong inhibitory effects on the Ca^2+^ response (IC_50_ < 2~10 μM). At present, we do not know the reason why the procedures used to detect TRPV2 activity gave different results. One likely possibility is that lipophilic agonist 2-APB may modulate the membrane integrity or interaction of TRPV2 with membrane lipids more strongly in a single cell experimental setting (such as patch clamp) in comparison with a cultured cell monolayer, which may offset the inhibition by other lipophilic chemical compounds. Indeed, similar to our observation, the effectiveness of other TRPV2 blockers was much reduced when high concentrations of 2-APB were used [21]. Furthermore, while tranilast (100 μM) did not inhibit channel activity when 200 μM 2-APB was used (data not shown), it blocked the pressure-induced inward current in TRPV2-transfected HEK293 cells [33].

We reported that TRPV2 accumulated in the sarcolemma of heart muscle cells and skeletal muscles cells in patients with DCM and muscular dystrophy, respectively [16, 19]. In addition, it has been reported that TRPV2 is involved in myotubes from patients with Duchenne muscular dystrophy [34]. Thus, specific inhibitors of TRPV2 could potentially be effective treatments for patients with various degenerative and hereditary diseases affecting muscles. An interesting aspect of TRPV2 as a therapeutic target is that it can be blocked using 2 fundamentally different strategies: by inhibiting sarcolemmal accumulation or channel activity. With regard to inhibition of sarcolemmal accumulation, we previously reported that reduction of sarcolemmal TRPV2 by overexpression of its amino terminal domain prevented cardiac dysfunction and DCM progression in animal models, while enhancing survival [19]. In addition, the present study provides evidence for the therapeutic potential of TRPV2 channel inhibition. A3 A48, A63, B6, and lumin blocked the progression of DCM in J2N-k hamsters, likely due to inhibition of Ca^2+^ influx through TRPV2; thus, TRPV2 may be an upstream factor in abnormal Ca^2+^ handling. Recently, genetic ablation of TRPV2 was reported to reduce heart performance [30] and result in abnormal cardiac structure and function [35]. These studies suggest that TRPV2 plays an essential role in maintaining normal cardiac structure and function. However, while TRPV2 inhibitors prevented cardiac dysfunction in DCM hamsters, they had no obvious detrimental effects on isolated normal cardiomyocytes or healthy control animals (Y. Iwata, *et al*., unpublished observation). Thus, TRPV2 inhibitors would not be expected to exert detrimental effects during limited administration periods. Further studies are required to assess the therapeutic uses of TRPV2 inhibitors.

In this study, we identified several TRPV2 inhibitors. Lumin has antioxidant, antitumor, and immunopotentiating effects [25, 36, 37]. Here, lumin was found to have a protective effect against DCM. Lumin has clinical potential because it is commercially available as an immunostimulant and is apparently without side effects. Tranilast is also available to patients as an anti-inflammatory and immunomodulatory compound and could also have clinical potential as a treatment for patients with DCM. To this end, novel inhibitors derived from tranilast may also have high therapeutic potential owing to their relatively low effective doses.

## MATERIALS AND METHODS

### Animal experiments

Male δ-SG-deficient cardiomyopathic J2N-k and age-matched wild-type J2N-n hamsters (9 weeks old) were purchased from Japan SLC, Inc. (Shizuoka, Japan). Tranilast was ingested mixed with feed (300 mg/kg/day) as described previously, whereas other drugs (Figure 1) were administered orally in the drinking water at doses of 30 mg/kg (except lumin) per day for 3 weeks. In case of lumin, we first performed dose-dependent experiment at dose of 1,10 and100 mg/kg per day for 3 weeks, in order to determine the effective dose. And then we repeated experiments at an effective dose (10 mg/kg) for 3 weeks. After drug treatment, serum cardiac troponin (cTN)-I was measured and echocardiography analysis was performed. Histochemistry of cardiac muscles was performed as previously described [38]. All animal experiments were performed according to the Guidelines for Animal Experimentation of the National Cerebral and Cardiovascular Center Research Institute.

### Histology

Ventricles were fixed in phosphate-buffered saline containing 10% formalin and embedded in paraffin. Serial sections were cut at a thickness of 5 μm and stained with Masson's trichrome stain for morphological analysis. Sections were viewed under a BX41 light microscope (Olympus, Tokyo, Japan). Images were acquired with an FX380 digital camera (Olympus) and analysed using an FLVFS-LS imaging system (Flovel, Tokyo, Japan).

Fibrosis was assessed by measuring Masson's trichrome-positive areas. Briefly, colour images were converted to binary form by setting the threshold value so as to detect only blue-stained fibrotic areas, which were summed and reported as a percentage of the total.

### Echocardiography

Cardiac function was evaluated by echocardiography using a Sonos5500 ultrasound system (Hewlett-Packard, Palo Alto, CA, USA) with a 12-MHz transducer and M-mode imaging. The animals were sedated with tribromoethanol (350 mg/kg by intraperitoneal injection) during the process.

### Reagents

Cariporide was a gift from Sanofi-Aventis (Bridgewater, NJ, USA). Poloxamer 188 (Exocorpol) was purchased from Midori Juji Co., Ltd. (Osaka, Japan). Nifedipine, streptomycin, 2-APB, 2-[(4-methoxyphenyl)carbonylamino]-3-methylbenzoic acid (A48) (S770000), and diltiazem were purchased from Sigma-Aldrich (St. Louis, MO, USA). Tranilast was obtained from Kissei Pharmaceutical (Matsumoto City, Japan). SKF96365 and ionomycin were obtained from Calbiochem (La Jolla, CA, USA). Ruthenium red was obtained from Wako Pure Chemical Industries Ltd. (Osaka, Japan). 2-[(2E)-3-(3-Chloro-4,5-dimethoxyphenyl)-2-cyanoprop-2-enoylamino]benzoic acid (A3) (NATR020256) and 1-{2-[3-((1E)-2-(1,2,4-triazol-4-yl)-2-azavinyl)indolyl]ethoxy}-3-methoxybenzene (B6) (NATR030155) were purchased from Vitas-M Laboratory, Ltd. (Apeldoorn, the Netherlands). (2E)-3-(3,4-Dimethoxyphenyl)-N-(2-nitrophenyl)prop-2-enamide (A63) (OSSK-422912), 4,5-dimethoxy-2-(phenylureido)benzoic acid (A65) (OSSK-423626), and 1-indolizin-2-yl-4-methoxybenzene (B33) (OSSK-004571) were obtained from Princeton University. 4,4′-[3-[2-[1-ethyl-4(1H)-quinolinylidene]ethylidene]-1-propene-1,3-diyl]bis(1-ethylquinolinium) diiodide (lumin) was kindly provided by Hayashibara Biochemical Laboratories (Okayama, Japan). Fura-2-acetoxymethylester (AM) was obtained from Dojindo Laboratories (Kumamoto, Japan). All chemicals used in the study were of the highest available purity.

### Molecular biology

Plasmid constructs of TRPV2, TRPV1, and transient receptor potential cation channel, subfamily C, member 1 (TRPC1) were generated using a PCR-based strategy from full-length cDNA sequences of mTRPV2 and hTRPV2, hTRPV1, and hTRPC1 cloned into the pIRES expression vector (Clontech Laboratories, Mountain View, CA, USA).

### Cell culture

Myocytes were cultured from enzymatically dissociated muscles of J2N-n and J2N-k hamsters as previously described [39]. Briefly, satellite cells were obtained from the gastrocnemius muscle using an enzyme cocktail containing 0.5 mM CaCl_2_. After enrichment of myoblasts by preplating over several rounds, cells were seeded in culture dishes. Two days after seeding, the culture medium was replaced with Dulbecco's modified Eagle's medium (DMEM) containing 2% horse serum to induce myotube formation. Myocytes were analysed 2–4 days after the start of fusion.

HEK293 and CHO cells were maintained in DMEM (containing 25 mM NaHCO_3_ and supplemented with 7.5% (v/v) foetal calf serum) and transfected with cDNA using Lipofectamine^®^ 2000 (Invitrogen). Stable clones were isolated by puromycin selection (Nacalai Tesque, Inc., Kyoto, Japan).

### [Ca^2+^]_i_ measurement

[Ca^2+^]_i_ was measured by a ratiometric fluorescence method as previously described [17]. Briefly, HEK293 cells were loaded with 4 μM fura-2-AM for 30 min at 37° C and maintained in balanced salt solution (BSS) (146 mM NaCl, 4 mM KCl, 2 mM MgCl_2,_ 0.5 mM CaCl_2_, 10 mM glucose, 0.1% bovine serum albumin, and 10 mM HEPES/Tris, pH 7.4). Fura-2-AM fluorescence was measured using an Aquacosmos fluorescence image processor (Hamamatsu Photonics, Hamamatsu, Japan). The time-course data are presented as the ratio of fluorescence at an excitation wavelength of 340 nm to that at 380 nm. The increase in the fluorescence ratio induced by the TRPV2 agonist 2-APB or high extracellular Ca^2+^ (ΔR) was normalized to the initial ratio (R_0_) before stimulation. The fluorescence relative ratio (ΔR/R_0_) was plotted as the summary data in the fluorescence imaging analysis.

For screening, cells were plated in 96-well plates and the fura-2-AM fluorescence ratio at excitation wavelengths of 340 nm and 380 nm was monitored using a GENios Pro microplate reader (Tecan, Research Triangle Park, NC, USA) or POLARstar Omega (BMG Labtech, Germany). Cells were stimulated in BSS containing 5 mM CaCl_2_. For stimulation with 2-APB (0.5 mM), the pH was adjusted to 6.5. All Ca^2+^ measurements were carried out at room temperature. Because the experimental conditions (filter setting, slit length, etc.) for the microplate reader analysis were different from those used in the fluorescence imaging analysis, these experiments produced different 340/380 nm fluorescence ratios. For simplicity, the results are presented as normalized values (% maximum) from the microplate reader experiment.

### Electrophysiology

TRPV2 currents were recorded under voltage-clamp conditions by the whole-cell patch-clamp technique [40]. Patch pipettes (2–5 MΩ) were filled with an intracellular solution containing 110 mM K-aspartate, 30 mM KCl, 10 mM NaCl, 1 mM MgCl_2_, 10 mM BAPTA, 10 mM HEPES, and 3 mM MgATP (adjusted to pH 7.2 with KOH). The extracellular solution contained 145 mM NaCl, 5 mM KCl, 1.5 mM MgCl_2_, 1 mM EGTA, 10 mM HEPES, and 10 mM glucose (pH was adjusted to 6.5 with NaOH). Because TRPV2 was reported to desensitize in the presence of extracellular Ca^2+^ [41], we used nominally Ca^2+^-free medium containing 1 mM EGTA to prevent desensitization. In the experiments confirming the pH dependence of the response, the pH was adjusted to 7.4. In the whole-cell recordings, the membrane potential of the cell was held at −60 mV, while voltage ramps of 400 ms from −100 mV to +60 mV were applied every 2 seconds. Currents through the electrode were recorded at 5 kHz and filtered at 2 kHz by an Axopatch 200B amplifier (Molecular Devices, LLC, Sunnyvale, CA, USA). The junctional potential was −11.7 mV. We did not compensate for the junctional potential in the calculations.

For the measurement of L-type Ca^2+^ currents, human Cav1.2/β2/α2δ1 calcium channel expressing CHO cells were purchased from ChanTest (Cleveland, OH, USA). The holding potential was held at −80 mV, while depolarizing pulses of 0 mV for 200 milliseconds were applied every 10 seconds. The composition of the extracellular solution was as follows: 145 mM NaCl, 4 mM KCl, 10 mM CaCl_2_, 10 mM HEPES, and 10 mM glucose (adjusted to pH 7.4 with NaOH). The composition of the intracellular solution was as follows: 112 mM CsCl, 27 mM CsF, 2 mM NaCl, 8.2 mM EGTA, 10 mM HEPES, and 4 mM MgATP (adjusted to pH 7.2 with CsOH). The data were analysed and plotted with Clampfit 9 (Molecular Devices, LLC, Sunnyvale, CA, USA). All experiments were performed at temperatures between 22° C and 25° C.

### Application of mechanical stretch to myocytes

Mechanical stretch was applied to myocytes using a chamber described in a previous study [42]. A silicon chamber with a transparent bottom (200 μm thickness) was attached to a stretching apparatus (NS-300; SCHOLAR-TEC Co., Osaka, Japan) driven by a computer-controlled stepping motor. Cells were allowed to attach to the chamber bottom for the indicated times. A constant, monoaxial sinusoidal stretch was applied from 5% to 20% elongation at 1 Hz, producing uniform elongation of the silicon membrane across the membrane area. Experiments were performed at 25° C ± 1° C.

### *In silico* ligand screening

The Namiki 2004 chemical library (Namiki Shoji Co., Ltd., Tokyo, Japan) was used to select 350 compounds via ligand-based virtual screening [43]. The previously reported active ligands tranilast and SKF96365 were used as reference molecules. Chemical structures were represented as molecular descriptors using the DRAGONX v.1.2 program (Talete S.r.l., Milan, Italy). The 350 top-ranked compounds were selected based on their similarity to the reference molecules as measured by the Tanimoto coefficient.

### Assays and data analysis

Creatine phosphokinase (CK) activity in the medium was determined using an *in vitro* colorimetric assay kit (CK Test Kit; Wako Pure Chemical Industries, Ltd., Osaka, Japan) according to the manufacturer's protocol The cTN-I level in the serum was measured, while quantitative immunoblotting and immunocytochemistry were performed as previously described [38, 39]. Protein concentration was measured using the bicinchoninic acid assay (Pierce Chemical Co., Rockford, IL, USA) with bovine serum albumin as the standard. All histochemical and physiological analyses were performed by investigators blinded to the drug treatments. Unless otherwise stated, results are presented as the mean ± SD of at least 5 determinations. The unpaired Student's *t*-test and one-way ANOVA followed by Dunnett's test were used to analyse the data. Values of *P* < 0.05 (indicated as asterisks in the figures) were considered statistically significant. All cellular measurements were repeated at least 5 times. Representative data are shown.

## Abbreviations

A3: 2-[(2E)-3-(3-chloro-4:5-dimethoxyphenyl)-2-cyanoprop-2-enoylamino]benzoic acid
A48: 2-[(4-methoxyphenyl)carbonylamino]-3-methylbenzoic acid
A63: (2E)-3-(3:4-dimethoxyphenyl)-N-(2-nitrophenyl)prop-2-enamide
A65: 4:5-dimethoxy-2-(phenylureido)benzoic acid
AM: acetoxymethylester
2-APB: 2-aminoethoxydiphenylborate
B6: 1-{2- [3-((1E)-2-(1:2:4-triazol-4-yl)-2-azavinyl)indolyl]ethoxy} −3-methoxybenzene
B33: 1-indolizin-2-yl-4-methoxybenzene
BSS: balanced salt solution
cTN: cardiac troponin
[Ca^2+^]_i_: cytosolic (intracellular) Ca^2+^ concentration
CK: creatine phosphokinase
DCM: dilated cardiomyopathy
DMEM: Dulbecco's modified Eagle's medium
EF: ejection fraction
FS: fractional shortening
lumin: 4:4′-[3-[2-[1-ethyl-4(1H)-quinolinylidene]ethylidene]-1-propene-1:3-diyl]bis(1-ethylquinolinium) diiodide
LVDd: left ventricular end-diastolic dimension
LVDs: left ventricular end-systolic dimension
SG: sarcoglycan
SKF96365: 1-[β-[3-(4-methoxyphenyl)propoxy]-4-methoxyphenethyl]-1H-imidazole
tranilast: N-[3:4-dimethoxycinnamonyl]-anthranilic acid
TRPV: transient receptor potential cation channel: subfamily V (vanilloid)
TRPC: transient receptor potential cation channel: subfamily C (canonical).
